# C/Co_3_O_4_/Diatomite Composite for Microwave Absorption

**DOI:** 10.3390/molecules29184336

**Published:** 2024-09-12

**Authors:** Yan Liao, Dashuang Wang, Wenrui Zhu, Zhilan Du, Fanbo Gong, Tuo Ping, Jinsong Rao, Yuxin Zhang, Xiaoying Liu

**Affiliations:** 1 College of Materials Science and Engineering, Chongqing University, Chongqing 400044, China; 202309131277@stu.cqu.edu.cn (Y.L.); wangdashuang@cqu.edu.cn (D.W.); 202209131285@stu.cqu.edu.cn (W.Z.); 202109021015@cqu.edu.cn (Z.D.); 20212399@stu.cqu.edu.cn (F.G.); rjs@cqu.edu.cn (J.R.); 2 Beijing Spacecrafts, China Academy of Space Technology, Beijing 100194, China; pinto1218@163.com; 3 Military Installations Department, Army Logistics Academy of PLA, Chongqing 401331, China

**Keywords:** microwave absorption, diatomite, cobalt oxides, dielectric loss, magnetic loss

## Abstract

Transition metal oxides have been widely used in microwave-absorbing materials, but how to improve impedance matching is still an urgent problem. Therefore, we introduced urea as a polymer carbon source into a three-dimensional porous structure modified by Co_3_O_4_ nanoparticles and explored the influence of different heat treatment temperatures on the wave absorption properties of the composite. The nanomaterials, when calcined at a temperature of 450 °C, exhibited excellent microwave absorption capabilities. Specifically, at an optimized thickness of 9 mm, they achieved a minimum reflection loss (RL_min_) of −97.3 dB, accompanied by an effective absorption bandwidth (EAB) of 9.83 GHz that comprehensively covered both the S and Ku frequency bands. On the other hand, with a thickness of 3 mm, the RL_min_ was recorded as −17.9 dB, with an EAB of 5.53 GHz. This excellent performance is attributed to the multi-facial polarization and multiple reflections induced by the magnetic loss capability of Co_3_O_4_ nanoparticles, the electrical conductivity of C, and the unique three-dimensional structure of diatomite. For the future development of bio-based microwave absorption, this work provides a methodology and strategy.

## 1. Introduction

Electromagnetic waves (EMWs) are being used more and more widely, resulting in serious EMW pollution problems [1,2,3]. In order to solve this problem, the research and application of EMW-absorbing materials has gradually been emphasized [4]. EMW-absorbing materials can reduce the harm of electromagnetic radiation to human health and improve the stability of communication equipment operation [5,6]. In addition, the stealth properties of electromagnetic wave-absorbing materials can be used in military applications [7,8]. The core challenge in designing EMW-absorbing materials lies in optimizing impedance matching and enhancing the attenuation capabilities, which are primarily influenced by the material’s composition and structure [9,10].

Bio-based materials have significant advantages, such as being lightweight, environmentally friendly, and renewable [11,12]. Therefore, they are expected to play an important role in EMW protection and electromagnetic pollution control in the future [13,14]. The microwave absorption of the material can be effectively improved through innovative structural design [15]. Numerous studies have reported that the cavity and pore structure of materials optimize impedance matching and increase multiple reflections [16,17,18]. For example, Wang et al. [19] prepared ZnO-doped magnetic graphene composite materials. The microwave absorption characteristics were enhanced by constructing rich microinterfaces and cavity structures. At a frequency of 8.96 GHz and a thickness of 1.81 mm, the RL_min_ reached −53.96 dB. Yang et al. [20] fabricated nitrogen-doped hollow carbon microspheres (N-HCMs), with a reflection loss of −61.3 dB at a matched thickness of 1.45 mm. Therefore, diatomite, as a biological material with a natural three-dimensional porous structure, has unique advantages when applied as an EMW-absorbing material [21,22]. For example, Wang et al. [23] used the hydrothermal method for the diatomite surface deposition of nickel cobalt LDH NW and then, through calcination, it was transformed into nickel and cobalt metal oxide and, finally, into etching composites. The results show that the RL_min_ at 7.1 mm was −82 dB and the EAB at 8 mm was 9.53 GHz. Zhu et al. [24] synthesized a Ni_x_S_y_@diatomite composite with excellent performance, the RL_min_ was −33.61 dB when the matching thickness was 1 mm. Li et al. [25] prepared MnFe_2_O_4_/rGO/diatomite cladding structured composites. The composite’s RL_min_ was −76.56 dB at 7.9 GHz, an EAB of up to 3.61 GHz, at a thickness of 2.485 mm. Therefore, by taking advantage of the naturally occurring porous three-dimensional structure of diatomaceous earth, it is possible to optimize impedance matching through structural modification and to combine it with lossy media, such as carbon and magnetic nanoparticles, in order to confer stronger wave-absorbing properties. Co_3_O_4_ magnetic nanoparticles are expected to be high-performance microwave-absorbing materials [26,27]. The spinel crystal structure of Co_3_O_4_, consisting of Co^2+^ and Co^3+^ located in the tetrahedral and octahedral interstitials, facilitates interfacial polarization [28,29]. In addition, carbon, with its excellent electrical conductivity and stability, provides the composite with outstanding electromagnetic properties and thermal stability [30,31]. The electrical conductivity of carbon material provides the composite material with an excellent electron transport channel [32,33]. Such material can significantly improve the electromagnetic properties of composites and is a big step forward in the research on EMW materials. For example, Han et al. [34] prepared ternary WSe_2_@CNTs/Co_3_O_4_ nanocomposites, with an RL_min_ of up to −54.09 dB and an EAB of up to 4.64 GHz. Chen et al. [35] prepared Co_3_O_4_/rGO composites. Composite material performs best when the RL is at −64.8 dB, it achieves the largest EAB of 9.63 GHz, with a matching thickness of 2.8 mm and 3 mm, respectively. However, single Co_3_O_4_ absorbers tend to be prone to agglomeration and have a high density [36]. Compositing it with other lightweight and dielectric materials can not only improve the agglomeration phenomenon, but also reduce its mass [37,38]. However, there is no systematic study on the application of diatomite nanocomposites decorated with C and Co_3_O_4_ nanoparticles for EMW absorption.

In this study, Co(OH)_2_ and diatomite were composited using a simple hydrothermal method and then mixed with polymeric urea, and carbon film-coated Co_3_O_4_/diatomite composites were successfully synthesized by in situ pyrolysis and polymerization of the carbon film in an inert atmosphere. Highly efficient microwave absorption was achieved through improved impedance matching by the carbon film on the surface and interfacial polarization of the multicomponent. In order to compare the effect of the pyrolysis temperature on the wave absorption performance, three different pyrolysis temperatures were used to tune the electromagnetic parameters. The nanomaterials, when calcined at a temperature of 450 °C, exhibited excellent microwave absorption capabilities. Specifically, at an optimized thickness of 9 mm, they achieved a minimum reflection loss (RL_min_) of −93.3 dB, accompanied by an EAB of 9.83 GHz that comprehensively covered both the S and Ku frequency bands. Therefore, this work provides insight into the construction of high-performance and low-cost microwave-absorbing materials.

## 2. Results and Discussion

**Figure 1:**
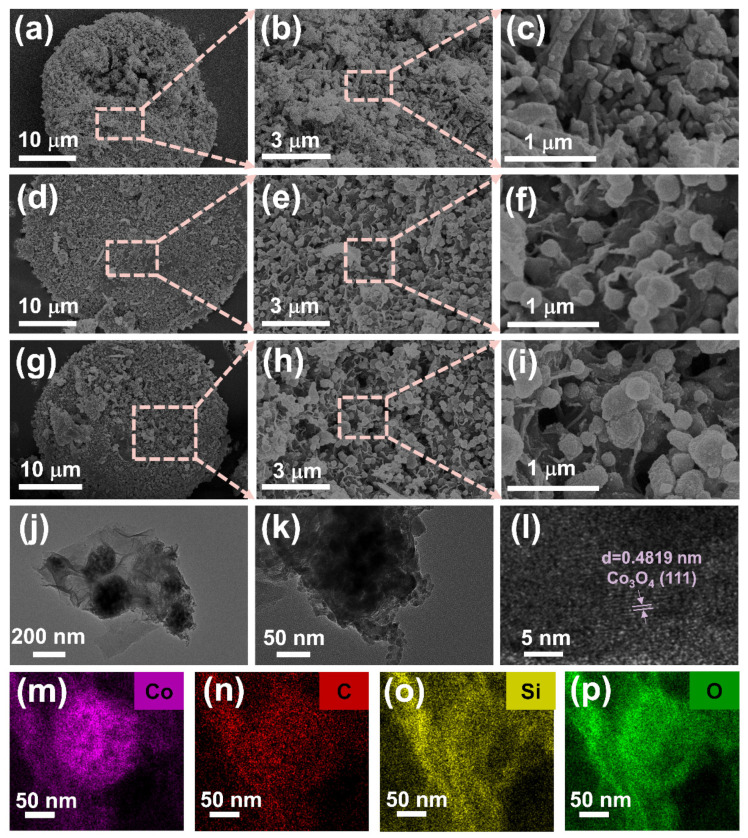
(**a**–**c**) SEM images of C/Co_3_O_4_/De-300, (**d**–**f**) C/Co_3_O_4_/De-450, and (**g**–**i**) C/Co_3_O_4_/De-600; (**j**–**l**) TEM images of C/Co_3_O_4_/De-600; and (**m**–**p**) HAADF-EDS element mapping images of Co, C, Si, and O of C/Co_3_O_4_/De-450.

SEM and TEM were used to characterize the microscopic topography and elemental distribution of the prepared composites, as shown in Figure 1. First, Co(OH)_2_/De precursors with a coral-like surface morphology were obtained using the hydrothermal method (as shown in Figure S1). After carbonization at 300, 450, and 600 °C in a nitrogen atmosphere, diatomite wrapped in Co_3_O_4_ was obtained, which was denoted as C/Co_3_O_4_/De-300, C/Co_3_O_4_/De-450, and C/Co_3_O_4_/De-600, respectively. The composites calcined at 300 °C are shown in Figure 1a–c, and the images at different magnifications show that the Co_3_O_4_ loaded on diatomite is in the shape of short rods and the loading is uneven. As shown in Figure 1d–f, the microscopic morphology of C/Co_3_O_4_/De-450 is granular and the Co_3_O_4_ nanoparticles are uniformly attached to the diatomite. The transformation from short rods to granules may be due to the sintering effect at high calcination temperatures [39]. Meanwhile, the loading of Co_3_O_4_ onto diatomite not only preserves the original porous structure of diatomite, but also increases the surface roughness of diatomite, and the unevenness of the surface tends to cause scattering loss [40].

From Figure 1g–i, it can be seen that C/Co_3_O_4_/De-600 and C/Co_3_O_4_/De-450 have similar surface morphology, but due to the high calcination temperature, the Co_3_O_4_ nanoparticles produce more violent agglomeration, which seriously affects the absorbing performance [41,42]. As shown in Figure 1j,k, the spherical Co_3_O_4_ is uniformly loaded onto the surface of diatomite. From Figure 1j, we can see the obvious lining degree, which we infer is a carbon film. Furthermore, through the elemental mapping diagrams (Figure 1m–p), we clearly observed the presence of Co, C, Si, and O elements and their distribution in the composites. In particular, the elemental distribution of Co is shown to be circular (Figure 1m), while the elemental distribution of carbon is dispersed (Figure 1n), proving the successful encapsulation of the carbon film. As can be seen from Figure 1l, the crystal plane spacing of 0.4819 mm corresponds to the (111) crystal plane of Co_3_O_4_. The successful synthesis of Co_3_O_4_ was confirmed.

**Figure 2:**
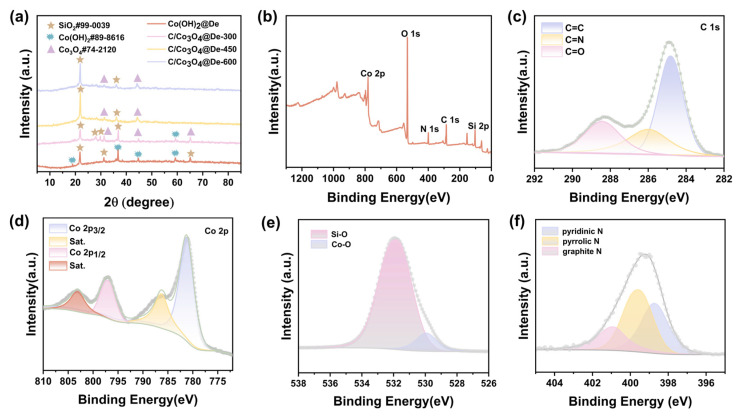
(**a**) XRD patterns and (**b**) XPS spectra of full survey scan, (**c**) C 1s spectrum, (**d**) Co 2p spectrum, (**e**) O 1s spectrum, and (**f**) N 1s spectrum of C/Co_3_O_4_/De-450.

Figure 2a characterizes the crystal structure of Co(OH)_2_/De and C/Co_3_O_4_/De by X-ray diffraction (XRD) analysis. In this pattern, the diffraction peaks at 21.9° and 28.28° clearly indicate the presence of the (101) and (111) crystal planes of SiO_2_ (JCPDS 99-0039). Using X-ray photoelectron spectroscopy (XPS), we explored the chemical composition of C/Co_3_O_4_/De-450 in depth, and the results are shown in Figure 2b–f. Specifically, the data in Figure 2b reveal the presence of Co, O, N, C, and Si within the sample, a finding that is consistent with the previous results obtained through the HAADF-EDS analyses in Figure 1m–p and Figure S3, which verifies the accuracy of the elemental composition. The content of each element can be obtained semi-quantitatively through the peak intensity (Table S1). The C 1s in the material is derived from organic matter in diatomite, and the three peaks at 288.4, 286.1, and 284.8 eV in the high-resolution C 1s spectrum (Figure 2c) are associated with C=O [43], C-N [44], and C-C/C=C [45], respectively. In Figure 2d, two characteristic peaks with binding energies at 803.2 eV and 797.2 eV, respectively, can be observed, which are attributed to the Co 2p1/2 orbital and its accompanying satellite peaks. Meanwhile, the other two peaks with binding energies at 781.3 eV and 786.3 eV correspond to the Co 2p3/2 orbital and its satellite peaks, separately. The results show that C/Co_3_O_4_/De exist in high spin Co^2+^ [46,47,48]. Figure 2e depicts the O-1s high-resolution spectrum, which exhibits two peak at 531.9 eV and 529.0 eV, indicative of the Si-O bond and Co-O band [49]. The presence of oxygen defects enhances the electrical conductivity, promotes efficient redox reactions, and ultimately contributes to the microwave absorption performance [50,51]. In the N 1s spectrum (Figure 2f), doped N shows three morphologies, pyridine-N (398.7 eV), pyridine-N (399.6 eV), and graphite-N (401.0 eV). In Figure S2, the peak at 102.7 eV binding energy is attributed to Si 2p in the diatomite. The above results show that Co(OH)_2_/De and C/Co_3_O_4_/De with the desired structure and morphology can be successfully prepared by a combination of the hydrothermal method and high-temperature pyrolysis.

**Figure 3:**
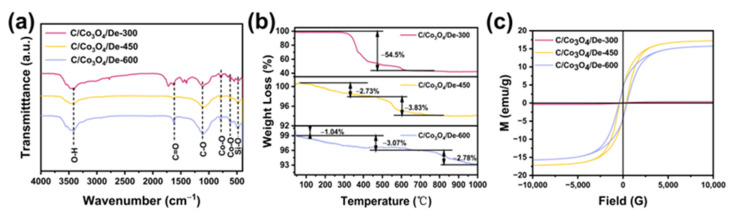
(**a**) FTIR spectra, (**b**) TGA, and (**c**) magnetic hysteresis loops for C/Co_3_O_4_/De-300, C/Co_3_O_4_/De-450, and C/Co_3_O_4_/De-600.

FTIR is used to study the type of functional groups in the sample. Figure 3a shows the FTIR spectrum in the wavelength range of 0–4000 cm^−1^. The absorption peak at 3414 cm^−1^ is related to the tensile vibration of O-H [52]. The peaks at 1617 cm^−1^ and 1115 cm^−1^ are C=O and C-O, respectively [53]. The typical peaks at 620.15 cm^−1^ and 782.09 cm^−1^ are Co-O for Co_3_O_4_, respectively [54,55]. The absorption peak at 479.1 cm^−1^ is considered to be the tensile vibration of the Si-O bond [56]. To evaluate the thermal stability of C/Co_3_O_4_/De composites, the samples were subjected to thermogravimetric analysis in an air atmosphere, and the results are shown in Figure 3b. Mass loss mainly occurs in two stages: 100–450 °C and 450–750 °C. When the temperature increases, the moisture and hydrogen bonds in the material are lost, causing the mass loss during the first stage. As the temperature increases further, the organic matter in the diatomite will be consumed, which is responsible for the mass loss during the second stage [57]. The mass loss of C/Co_3_O_4_/De-450 is only 2.73% at 100–450 °C. At a high temperature of 1100 °C, the mass reduction of the composite is only about 6% of its original mass. It can be seen that the material has excellent thermal stability and can adapt to EMW absorption application under high temperature conditions. Figure 3c shows the hysteresis loops of the samples, all the C/Co_3_O_4_/De samples exhibit soft magnetic properties, with saturation magnetization of 0.34, 17.17, and 15.81 emu/g for the C/Co_3_O_4_/De calcined at 300, 450, and 600 °C, respectively. The corresponding coercivity values are 58.66, 359.53, and 439.76 Oe, respectively. The reason for the difference in magnetic properties of the samples may be that the heat treatment temperature affects the arrangement of the magnetic moments, the structure, and the morphology of the magnetic domains in the material. In addition, there was no significant difference between the three sample conductivity values (Table S2). The results show that the main reason for the different performance of the three samples is the difference in the magnetic loss.

**Figure 4:**
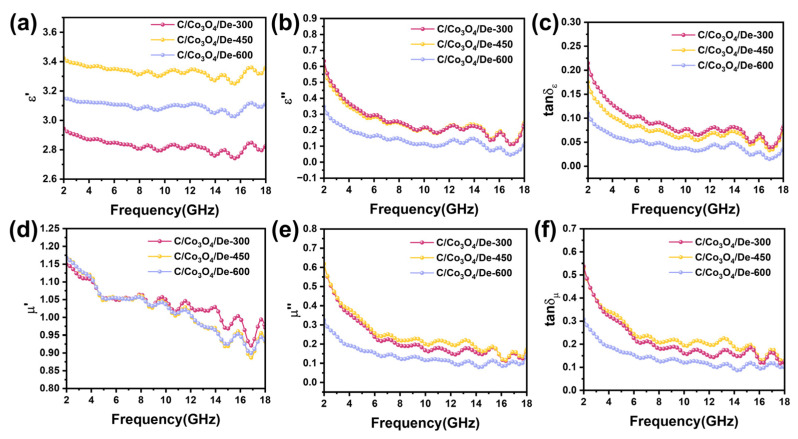
(**a**) Real part of permittivity, (**b**) imaginary part of permittivity, and (**c**) dielectric loss tangent plots of all the samples; (**d**) real part of permeability, (**e**) imaginary part of permeability, and (**f**) dielectric loss tangent plots of all samples.

The complex permittivity ($\varepsilon_{r}=\varepsilon \prime -j\varepsilon \prime \prime$) and complex permeability ($\mu_{r}=\mu \prime -j\mu \prime \prime$), which describe the response properties of materials in the microwave frequency band, have a profound effect on the microwave absorption properties of wave absorbers. They can be employed to elucidate the attenuation mechanisms by which wave-absorbing materials convert incident EMW energy into heat or other forms of energy. Figure 4a,b presents the complex permittivity of C/Co_3_O_4_/De-300, 450, 600 composites across the 2–18 GHz frequency range. The real and imaginary parts of the complex dielectric constant (ε″ and μ″) represent the role of the material in the storage and loss of electromagnetic energy under the action of an electric field, respectively. Notably, C/Co_3_O_4_/De-450 exhibits the highest ε′, ranging from 3.0 to 3.5, balancing the dielectric loss and impedance matching. The ε′ values for all the samples show multiple fluctuations in the 12–18 GHz range, suggesting significant polarization loss during EMW attenuation. This is likely due to interfacial polarization from the surface charge accumulation on the Co_3_O_4_ and diatomite heterojunction, and dipole polarization from N-O and C-O functional groups introduced during calcination. For ε″, C/Co_3_O_4_/De-450 and C/Co_3_O_4_/De-300 follow similar trends and have higher values than C/Co_3_O_4_/De-600, possibly due to the fragmentation of Co_3_O_4_ and diatomite during high-temperature calcination, impacting EMW transmission. The thermogravimetric curves and wave-absorbing properties further confirm this. The ε″ values for all the samples exhibit a disordered trend with multiple spurious peaks, stabilizing only in the 2–5 GHz range. This behavior may be due to the synergistic effect of the space charge and orientation polarization caused by the unique evenly distributed porosity on the surface of diatomite coupled with the interfacial polarization generated by the middle cavity structure and surface-loaded transition metal oxides.

The dielectric loss (${\tan\delta }_{\varepsilon }=\;\varepsilon \prime \prime /\;\varepsilon \prime$) is often used to characterize the dielectric loss capability of an absorber. Figure 4c shows that the dielectric loss capability of C/Co_3_O_4_/De-450 is comparable to that of C/Co_3_O_4_/De-300, following the same trend as ε″. Figure 4d,e illustrates the complex permeability of the three samples within the 2–18 GHz frequency range. The μ′ value tends to weaken in the low frequency band, the possible reason is that the rod-like Co_3_O_4_ will form a conductive grid, and the eddy current loss will be suppressed to a certain extent during the transmission of electrons. The presence of ferromagnetic resonance and eddy current effects results in significant fluctuations in the μ″ curve over the entire frequency range, with the appearance of several resonance peaks. It is noteworthy that the pronounced fluctuations in μ″ at elevated frequencies are attributable to exchange resonance, which serves to augment the magnetic loss capacity in wave-absorbing materials. Notably, the large oscillations in μ″ at high frequencies are due to exchange resonance, which contributes to magnetic loss in wave-absorbing material. Interestingly, the magnetic loss tangent has a distinct peak at 15 GHz, with formants at high frequencies, representing natural resonance. At the same time, it can be seen from Figure 4f that the dielectric loss tangent of the three samples all have some significant formant peaks, which can also be called polarization peaks. This behavior may be due to the effect of conduction loss and polarization relaxation on the loss capacity of the medium. The high conductivity of the C film is conducive to the migration of free electrons, resulting in a significant loss of conductivity. In addition, the abundant defects and vacancies in Co_3_O_4_ act as dipole polarization centers and promote polarization relaxation. In addition, the combination of Co_3_O_4_ and diatomite increases the polarization area and improves the interface polarization. Notably, ${\tan\delta }_{\mu }$ exceeds ${\tan\delta }_{\varepsilon }$ within the 2–18 GHz range, indicating that magnetic loss is the dominant form of EMW absorption in these materials.

**Figure 5:**
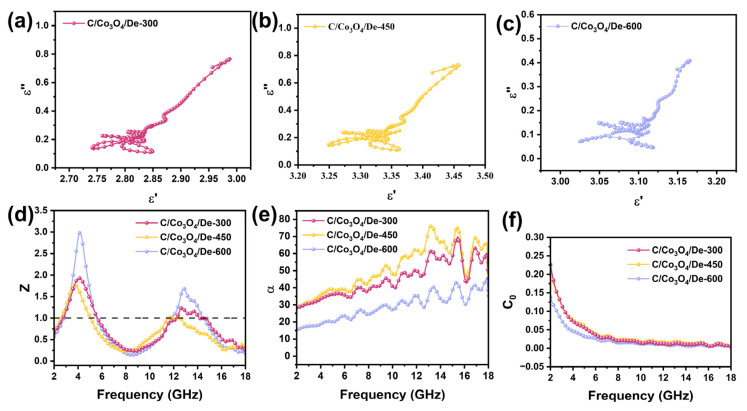
(**a**) Cole–Cole plots of C/Co_3_O_4_/De-300, (**b**) C/Co_3_O_4_/De-450, (**c**) C/Co_3_O_4_/De-600; plots of (**d**) impedance matching, (**e**) attenuation constants, and (**f**) eddy current loss of all the samples.

To further analyze the dielectric loss capability, we examine the dielectric constants and plot the Cole–Cole curves (Figure 5a–c) to elucidate the dielectric loss mechanisms. According to Debye’s theory, each semicircle in the curve corresponds to a relaxation process. Interestingly, we observe a variable number of semicircles in all three samples, indicating that the Debye relaxation process is prevalent in the prepared materials. The presence of multiple semicircles indicates the presence of multiple relaxation processes, and irregular curves suggest that the polarization loss of the materials is a combination of dipole polarization and dielectric polarization. These mechanisms are associated with the interface between diatomite and Co_3_O_4_ and the C film. Also, smooth tails are observed in all three samples, suggesting that conduction losses also have an effect on dielectric losses.

In order to further analyze the magnetic losses, we analyze the eddy current loss curves. The most common sources of magnetic losses are hysteresis, domain wall resonance, natural ferromagnetic resonance, and eddy current effects. In scenarios involving weak magnetic fields, hysteresis losses are generally insignificant. Conversely, domain wall resonance losses commonly manifest at significantly lower frequencies, namely the MHz range. Consequently, natural ferromagnetic resonance and eddy current effects are frequently regarded as the primary mechanisms responsible for energy losses in ferromagnetic absorbers operating within the gigahertz frequency range. The eddy current loss can be expressed as Equation (1) [58]:

(1) $$C_{0}=\mu \prime \prime {\left(\mu \prime \right)}^{-2}{\left(f\right)}^{-1}=2{\pi \mu }_{0}d^{2}\sigma$$

where σ (S/m) is the electrical conductivity and $\mu_{0}$ (H/m) is the vacuum permeability. If the sole cause of the reflection loss is the eddy current effect, then the magnitude of $C_{0}$ remains constant across different frequencies [59]. Figure 5f illustrates that the $C_{0}$ values remain stable between 8–18 GHz, indicating that eddy current losses are the primary mechanism responsible for magnetic losses. Furthermore, the $C_{0}$ values of the three samples continue to decrease within the frequency range of 2–8 GHz, a phenomenon that suggests that the eddy current effect is not the sole factor contributing to the energy loss and that natural ferromagnetic resonance may also play a significant role in the magnetic loss. This phenomenon can be explained as follows: (1) Co_3_O_4_, as a transition metal oxide, is an effective magnetic conductor, which, under an alternating magnetic field, forms a crosslinked network that enhances eddy currents and the skin effect; (2) the lower degree of amorphous carbon shells on the surface of Co_3_O_4_ prevents Co_3_O_4_ agglomeration and interrupts eddy currents, thereby inhibiting the skin effect to some extent and allowing the magnetic function of the Co_3_O_4_ shell layer to be fully utilized. The attenuation constant α (dB/m) can be derived from Equation (2), which employs the principles of transmission line theory, thus providing a quantitative measure of the wave-absorbing material’s capability to attenuate signals [60]:

(2) $$\alpha =\left(\sqrt{2}\pi f/c\right)\times \sqrt{\left(\mu \prime \prime \varepsilon \prime \prime -\mu \prime \varepsilon \prime \right)+\sqrt{{\left(\mu \prime \prime \varepsilon \prime \prime -\mu \prime \varepsilon \prime \right)}^{2}+{\left(\mu \prime \varepsilon \prime \prime +\mu \prime \prime \varepsilon \prime \right)}^{2}}}$$

where f is the frequency and c is the speed of light. As illustrated in Figure 5e, the plot depicts the relationship between the attenuation coefficient and frequency for each sample, demonstrating that the attenuation capacity is a fundamental aspect in evaluating the absorption performance of these materials. The attenuation constant exhibited by C/Co_3_O_4_/De-450 throughout the frequency range spanning from 2.0 to 18.0 GHz consistently surpasses that of C/Co_3_O_4_/De-300 and C/Co_3_O_4_/De-600, with a pronounced disparity observed in the higher frequency bands. This highlights the enhanced attenuation capacity of C/Co_3_O_4_/De-450 in relation to incoming EMW.

In addition to the attenuation coefficient, impedance matching (|$Z_{\mathrm{in}}/Z_{0}$|) is of paramount importance in assessing the efficacy of EMW-absorbing materials in terms of their absorption capabilities. Strong attenuation cannot directly translate into absorption capacity; the EMW must first enter the absorber material to exhibit a loss effect, known as impedance matching. Impedance matching explains the proportion of incident microwaves that penetrate the absorber surface and propagate within it. High-performance wave-absorbing materials should have a Z-value greater than 0.3, with a Z-value of 1 indicating free-space impedance, meaning the EMW can propagate freely [61]. In accordance with the design guidelines for wave-absorbing materials, it is imperative to achieve a |$Z_{\mathrm{in}}/Z_{0}$| value as close to 1 as feasible. This indicates that more EMW will enter the interior of the material and, thus, will be dissipated by various mechanisms. The |$Z_{\mathrm{in}}/Z_{0}$| versus the frequency is derived from Figure 5d. It can be observed that the curve of C/Co_3_O_4_/De-450 is closer to 1, which aligns with its exemplary EMW absorption performance. The other materials have larger differences in terms of their impedance matching values and poorer impedance matching performance, resulting in poorer EMW absorption performance.

The reflection loss characteristics RL (dB) of the three composite nanomaterial samples can be deduced from their respective complex permittivity and complex permeability using transmission line theory [62], as shown in Equation (3):

(3) $$\mathrm{RL}\left(\mathrm{dB}\right)=20\mathrm{lg}\left|\frac{Z_{\mathrm{in}}-Z_{0}}{Z_{\mathrm{in}}+Z_{0}}\right|$$

**Figure 6:**
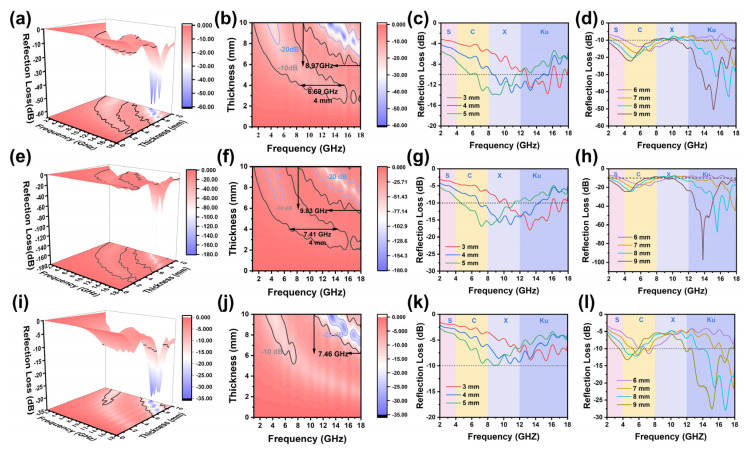
A 3D loss diagram, contour plot of RL with thickness and frequency (S, C, X and Ku bands), and frequency–loss 1D diagram of (**a**–**d**) C/Co_3_O_4_/De-300, (**e**–**h**) C/Co_3_O_4_/De-450, and (**i**–**l**) C/Co_3_O_4_/De-600.

$Z_{\mathrm{in}}$ is the normalized input impedance of the microwave absorbing layer. Figure 6 depicts three-dimensional plots of the reflection loss, two-dimensional planar plots, and one-dimensional plots at specific thicknesses, for the three samples. As the coating thickness increases, it is observed that the EAB frequency and peak reflection loss for all three samples shift towards the lower frequency region. This phenomenon is completely consistent with the one-quarter wavelength model in transmission line theory, which further verifies the reliability of the experiment and the authenticity of the data. In terms of absorption efficiency, all three samples exhibit enhanced reflection loss characteristics and higher high-frequency absorption. By adjusting the thickness, the absorption of almost the complete coverage of the full frequency band (S, C, X, Ku) can be achieved. It is evident that all three samples can absorb the incident EMW, but their specific performances vary significantly. As shown in Figure 6i,j, C/Co_3_O_4_/De-600 has almost negligible absorption at a thickness of 5 mm. However, when the thickness is increased to 9 mm, the sample achieves an RL_min_ of −27.8 dB, while the EAB band is extended to 7.24 GHz.

As illustrated in Figure 6e–h, the comprehensive performance of C/Co_3_O_4_/De-450 is demonstrably superior to that of the other samples. Ideal EMW absorbers should fulfill four criteria: a high RL, a wide EAB, light weight, and thin thickness. Among these factors, thickness and bandwidth are the key determinants of the adaptability of the wave-absorbing material in regard to portable device and aerospace applications. The EAB of C/Co_3_O_4_/De-450 reaches 7.53 GHz at a thickness of only 4 mm. The material is capable of covering the entire X band and part of the Ku band, with an RL_min_ of −16.4 dB. When the thickness is increased to 9 mm, the sample achieves an RL_min_ of −97.3 dB, while the EAB dramatically increases to 9.83 GHz. It exhibits effective absorption performance across the entire S and Ku band, thereby ensuring broadband coverage. This highlights the potential of this material as an EMW absorber.

The maximum RL of C/Co_3_O_4_/De-300 significantly decreases compared to C/Co_3_O_4_/De-450, but the EAB remains satisfactory. At 9 mm, it achieves an EAB of 8.97 GHz, with the RL_min_ decreasing to −50.1 dB (Figure 6a–d). Figure S4 compares the performance of the wave-absorbing materials. Obviously, the composite nanomaterials prepared in this study exhibit more significant EAB widths, as well as enhanced reflection loss capability. This strongly suggests the great potential of C/Co_3_O_4_/De-450 as an EMW-absorbing material that can flexibly adapt to the various demands placed on the wave-absorbing material’s performance in diverse environments.

In the field of microwave absorption, the evaluation of absorbers is dependent upon the assessment of several key parameters, including the reflection loss and response bandwidth. Therefore, we conducted a comparative assessment of C/Co_3_O_4_/De-450 with similar reported composites (Table S3). Excitingly, Co/C microspheres exhibited superior performance in regard to both the reflection loss and response bandwidth, affirming that nanocomposites based on C/Co_3_O_4_/De-450 biotemplates are promising candidates for next-generation EMW-absorbing materials.

**Figure 7:**
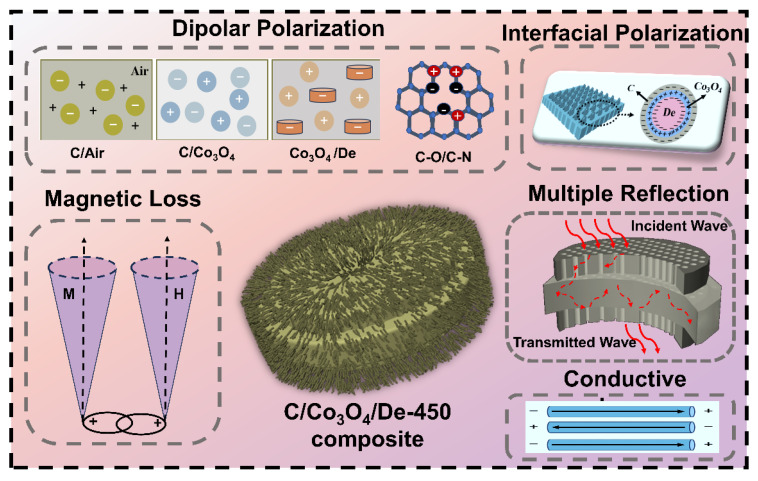
Schematic representation of EMW absorption diagram for C/Co_3_O_4_/De-450 composite.

Figure 7 proposes related microwave absorption mechanisms. Firstly, the microporous and cavity structures of diatomite facilitate the reflection, refraction, and scattering of EMWs. The periodic three-dimensional structure of diatomite provides ample propagation paths for incident microwaves, enhancing multiple reflections and scattering within the absorber, thereby boosting microwave absorption. Secondly, the C film formed during calcination and Co_3_O_4_ nanomaterials synergistically enhance impedance matching in C/Co_3_O_4_/De-450. Thirdly, the presence of disordered carbon, defects, and N-doped carbon on the surface of C/Co_3_O_4_/De-450 reduces crystallinity, as observed in the FTIR and XPS spectra. These defects and N-doped sites act as polarization centers, further enhancing dielectric loss. Finally, the differing media interfaces between materials facilitate the aggregation and transfer of additional free electrons to interfaces, promoting interfacial polarization and enhancing the conversion of EMWs into thermal energy dissipation. This contributes significantly to the excellent dielectric properties of C/Co_3_O_4_/De-450. The multipolar interfaces of the composites studied primarily originate from the boundaries between air and C, C and Co_3_O_4_, and Co_3_O_4_ and diatomite.

**Figure 8:**
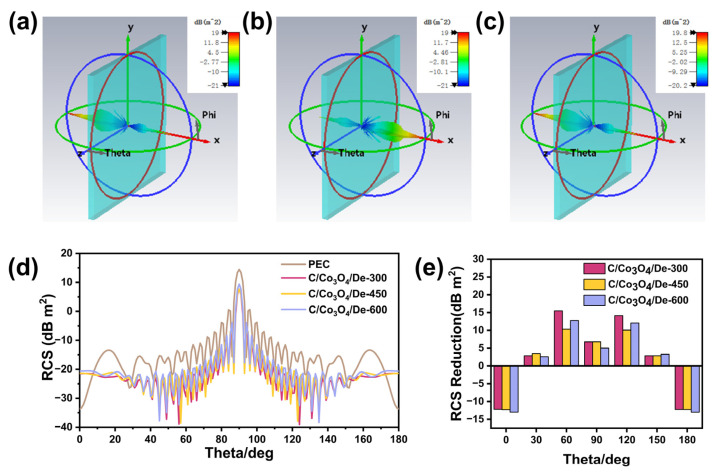
The CST simulation results for (**a**) C/Co_3_O_4_/De-300, (**b**) C/Co_3_O_4_/De-450, (**c**) C/Co_3_O_4_/De-600. (**d**) The simulated RCS curves of the PEC and C/Co_3_O_4_/De at a scattering angle of 0–180°. (**e**) The RCS reduction values (the RCS values of PEC minus that of the samples) for all the samples.

Wave-absorbing materials with small radar cross-section (RCS) values are considered to have good radar stealth capabilities. In order to investigate the EMW absorption performance of the prepared C/Co_3_O_4_/De materials under actual far-field conditions, we performed RCS simulations of the three composite nanomaterials using a computer simulation technique (CST). Figure 8a–c shows the three-dimensional intensity images of the reflected signals from the metal substrates (perfect electrical conductor, PEC) after coating C/Co_3_O_4_/De-300, C/Co_3_O_4_/De-450, and C/Co_3_O_4_/De-600, respectively. In contrast, the scattering signal of the C/Co_3_O_4_/De-450 sample is notably weak, indicating that it exhibits excellent absorption properties. Figure 8d illustrates the 1D RCS curves of the three model panels, with PEC panels in an angle range of 0–180°, and Figure 8e visualizes the RCS reduction values of the composite coatings for selected angles of incidence. All the samples showed a reduction in the RCS values, with C/Co_3_O_4_/De-450 exhibiting the strongest EMW absorption performance at any angle, with an RCS reduction of 19.1 dB m^2^ at 90°. The outcomes of the CST simulations are in alignment with the EMW absorption properties derived from transmission line theory, which provides a robust theoretical foundation for the practical implementation of EMW absorption in C/Co_3_O_4_/De composites.

## 3. Materials and Methods

### 3.1. Materials

The materials used in this experiment were cobalt nitrate hexahydrate (Co(NO_3_)_2_·6H_2_O), urea (CO(NH_2_)_2_), and diatomite, all of which were purchased from Shanghai Aladdin Biochemical Technology Co., Ltd., Shanghai, China. All drug products were analytical grade and required no further purification. The deionized water used in the experiment was made in the laboratory.

### 3.2. Preparation of Co(OH)_2_/Diatomite

The preparation of Co(OH)_2_/diatomite was carried out using the hydrothermal method. Typically, 1.3949 g (4.8 mmol) of Co(NO_3_)_2_·6H_2_O and 0.8 g of diatomite were dissolved in 300 mL of deionized water and stirred magnetically for 10 min at room temperature to obtain solution A. Secondly, 800 mg of urea was added to 300 mL of deionized water and stirred magnetically for 10 min at room temperature to obtain solution B. Finally, solution A and solution B were homogeneously mixed and transferred to a PTFE-lined stainless steel autoclave (100 mL) for 16 h in a rotary oven at 160 °C. The sample was allowed to cool naturally to room temperature, washed and filtered, and then dried in an oven at 70 °C for 20 min to obtain the Co(OH)_2_/diatomite sample. Further, the sample was named as Co(OH)_2_/De.

### 3.3. Preparation of C/Co_3_O_4_/Diatomite

**Figure 9:**
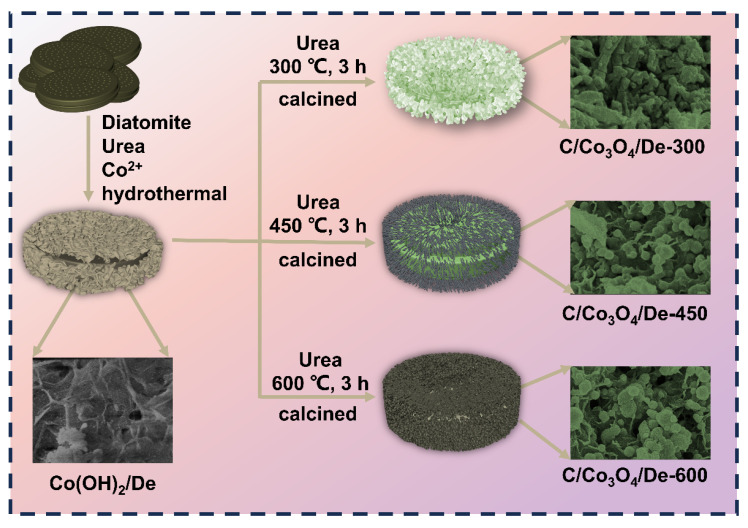
The synthesis process of C/Co_3_O_4_/De.

The Co(OH)_2_/De prepared during the previous step was evenly mixed with urea, according to a mass ratio of 1:5 and then calcined in a tube furnace. In a nitrogen environment, the sample was subjected to an increasing temperature at a speed of 5 °C/min to a certain temperature (300, 450, and 600 °C), and then remained at this temperature for 3 h. After natural cooling to room temperature, the final sample was obtained. Further, the samples were named as C/Co_3_O_4_/De-300, C/Co_3_O_4_/De-450, and C/Co_3_O_4_/De-600, respectively. The synthesis process of C/Co_3_O_4_/De is shown in Figure 9.

### 3.4. Characterization

Scanning electron microscopy (SEM, Zeiss Auriga, Jena, Germany) and transmission electron microscopy (TEM, Talos F200S, Waltham, MA, USA) were used to characterize the surface morphology and microstructure of the synthetic materials. The phase composition, element type, and valence state of the sample were obtained by X-ray powder diffraction (XRD, Rigaku Ultima IV, Tokyo, Japan, Cu-Kα) and Fourier transform infrared spectroscopy (FTIR, Nicolet iS50, Waltham, MA, USA). The valence states of C, O, Si, N, and Co in the surface area were measured by X-ray photoelectron spectroscopy (XPS, Thermo Fisher Scientific K-ALPHA, Waltham, MA, USA). The hysteresis loop at room temperature was measured with a vibrating sample magnetometer (VSM, LakeShore7404, Columbus, OH, USA). The resistivity of the sample was tested by a powder resistance tester (ST2722), using the four-probe method. The thermal stability of the sample was investigated using thermogravimetric analysis (TGA, Mettler 1100LF, Zurich, Switzerland) by recording the change in the sample mass from room temperature to 1100 °C. The electromagnetic parameters (dielectric constant and permeability) of the samples in the frequency range of 2–18 GHz were measured by a vector grid network analyzer (Agilent E5071c, Santa Rosa, CA, USA) using the coaxial method.

## 4. Conclusions

In summary, Co(OH)_2_/De was successfully synthesized using a hydrothermal method and then converted into the C/Co_3_O_4_/De composite by a calcination process. The surface morphology and electromagnetic parameters can be mediated by the heat treatment temperature. The results show that the RL_min_ is −97.3 dB and the EAB is 9.83 GHz at a matched thickness of 9 mm. The EAB covers the entire S and Ku bands. At a matched thickness of 3 mm, the RL_min_ is −17.9 dB and the EAB is 5.53 GHz. The Co_3_O_4_ nanoparticles have good synergistic effects with diatomite, and the 3D porous structure induces multiple reflections and scattering, dipole polarization and interfacial polarization is induced by the multilayer interfaces, as well as strong natural resonance and conduction loss. Therefore, the results of this study demonstrate the great potential of C/Co_3_O_4_/De composites as wave-absorbing materials and provide a direction for the future development of bio-based EMW-absorbing materials with greater absorption capacity and absorption range.

## Data Availability

Data are contained within the article and Supplementary Materials.

## Supplementary Materials

The following supporting information can be downloaded at: https://www.mdpi.com/article/10.3390/molecules29184336/s1, Figure S1: The scanning electron microscopy (SEM) images of Co(OH)2/De with different scales (a) 10 µm, (b) 5 µm and (c) 1 µm; Figure S2: High-resolution XPS spectra of Si 2p; Figure S3: HAADF-EDS element mapping images of (a) C/Co3O4/De-450 and (b) N of the C/Co3O4/De-450; Table S1: Elemental concentrations of C/Co3O4/De-450 obtained from XPS spectra; Table S2: Comparison of electrical resistivity and electrical conductivity of C/Co3O4/De-300, C/Co3O4/De-450, and C/Co3O4/De-600; Figure S4: Comparison of C/Co3O4/De-450 with other representative materials [63,64,65,66,67,68,69]; Table S3: Comparison of related absorber materials [63,64,65,66,67,68,69].
