# Supplementary material for: C/Co3O4/Diatomite Composite for Microwave Absorption
Source: Molecules. 2024 Sep 12;29(18):4336. doi: 10.3390/molecules29184336 (PMC11434343; doi:10.3390/molecules29184336)
Supplement: Supplementary file 1 [file molecules-29-04336-s001.zip › molecules-3175325-supplementary.pdf]

Supporting Information

C/Co_3O_4/Diatomite Composite for Microwave Absorption

Yan Liao [1,†], Dashuang Wang [1,†], Wenrui Zhu [1], Zhilan Du [1], Fanbo Gong [1], Tuo Ping [2], Jinsong Rao [1], Yuxin Zhang [1,*] and Xiaoying Liu [3,*]

[1] College of Materials Science and Engineering, Chongqing University, Chongqing 400044, China; 202309131277@stu.cqu.edu.cn (Y.L.); wangdashuang@cqu.edu.cn (D.W.); 202209131285@stu.cqu.edu.cn (W.Z.); 202109021015@cqu.edu.cn (Z.D.); 20212399@stu.cqu.edu.cn (F.G.); rjs@cqu.edu.cn (J.R.)

[2] Beijing Spacecrafts, China Academy of Space Technology, Beijing 100194, China; pinto1218@163.com

[3] Military Installations Department, Army Logistics Academy of PLA, Chongqing 401331, China

* Correspondence: zhangyuxin@cqu.edu.cn (Y.Z.); lxy_ctbu@163.com (X.L.)

† These authors contributed equally to this work.

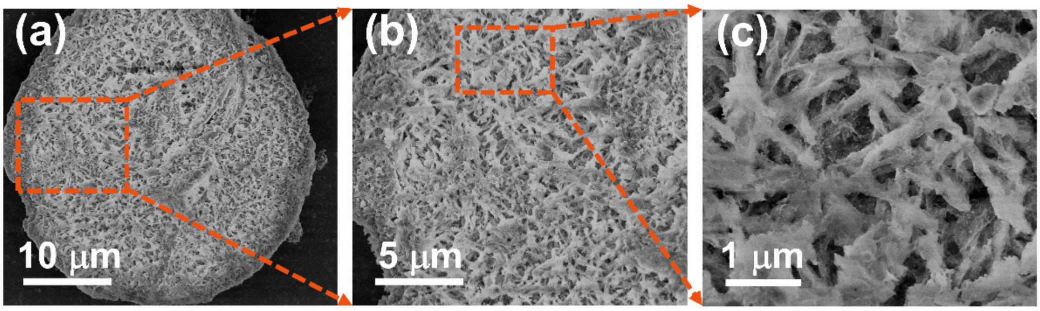

Figure S1. The scanning electron microscopy (SEM) images of $Co(OH)_2$/De with different scales (a)10 μm, (b) 5 μm and (c) 1 μm.

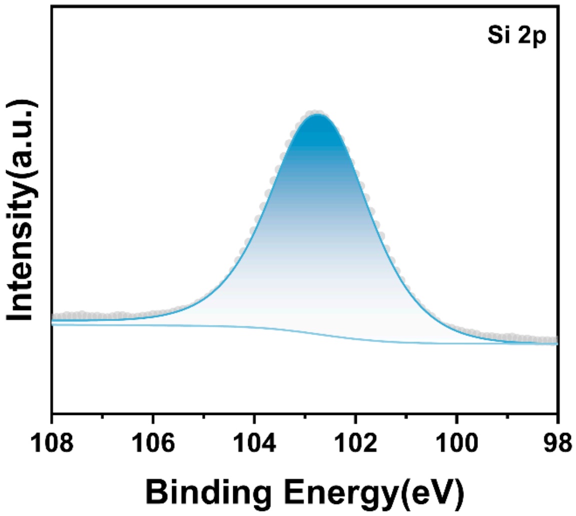

Figure S2. High-resolution XPS spectra of Si 2p

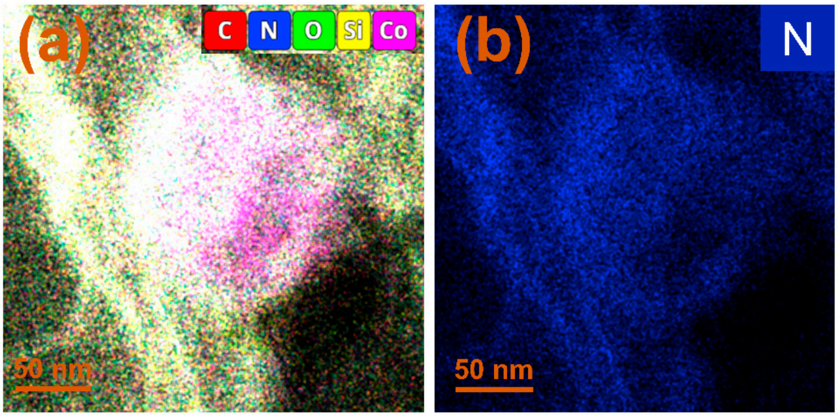

Figure S3. HAADF-EDS element mapping images of (a) C/Co_3O_4/De-450 and (b) N of the C/Co_3O_4/De-450

Table S1. Elemental concentrations of $C/Co_3O_4/De$-450 obtained from XPS spectra

Name	Area (P) CPS.eV	Atomic %	Weight %
O 1s	3456474.77	49.78	36.40
Si 2p	586526.85	20.35	26.11
Co 2p	3390402.06	16.21	26.03
C 1s	465478.50	9.67	8.90
N 1s	178235.46	4.00	2.56

Table S2. Comparison of electrical resistivity and electrical conductivity of $C/Co_3O_4/De$-300, $C/Co_3O_4/De$-450, and $C/Co_3O_4/De$-600.

Samples	Average electrical resistivity (μΩ cm)	Average electrical conductivity (S cm^{-1})
$C/Co_3O_4/De$-300	135838.889	7.36^{-6}
$C/Co_3O_4/De$-450	136611.111	7.32^{-4}
$C/Co_3O_4/De$-600	130238.889	7.68^{-4}

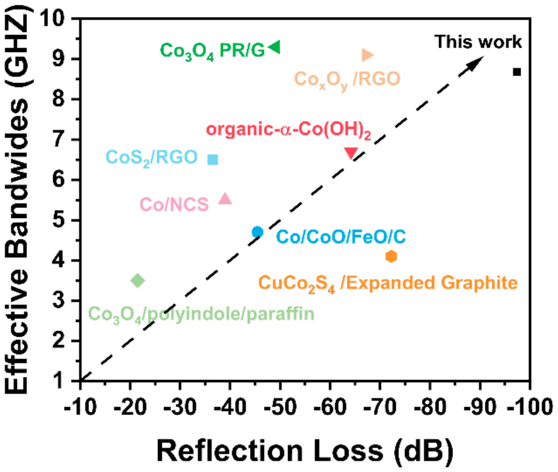

Figure S4. Comparison of $C/Co_3O_4/De$-450 with other representative materials.

Table S3. Comparison of related absorber materials.

Materials	RL_{min} (dB)	EAB (GHz)	Thickness (mm)	Reference
$CuCo_2S_4$@Expanded Graphite	-72.28	4.14	1.4	[1]
CoO/Co_3O_4	-67.2	9.1	2.62	[2]
Co3O4 PR/G	-49.1	9.3	4	[3]
Co_3O_4/polyindole/paraffin	21.5	3.5	2	[4]
organic-α-$Co(OH)_2$	-64.2	6.7	2.17	[5]
Co@NCS	-39	5.5	2.5	[6]
CoS_2/RGO	-36.5	6.5	2	[7]
C/Co_3O_4/De-450	-98.7 dB	9.52	9	This work
	-17.8 dB	6.45	3	This work

1. Tang Z.; Xu L.; Xie C.; Guo L.; Zhang L.; Guo S.; Peng J. Synthesis of $CuCo_2S_4$@Expanded Graphite with crystal/amorphous heterointerface and defects for electromagnetic wave absorption. *Nat. Commun.* **2023**, 14, 5951, https://doi.org/10.1038/s41467-023-41697-6.
2. Ding Z.; Du Z.; Liu Y.; Zhang Q.; Zhao Z.; Hou M.; Wang X.; Hassan Y. A.; Huang X.; Yue J.; Chen A.; Ma Y.; Lu S. Reduced graphene oxide loaded with rich defects CoO/Co_3O_4 for broadband microwave absorption. *Compos. Part B: Engineering*. **2023**, 249, 110403, https://doi.org/10.1016/j.compositesb.2022.110403.
3. Fu M.; Yu H.; Chen W. Construction of Co_3O_4 porous rod/graphene heterostructures toward strong and broadband microwave absorption applications. *Appl. Surf. Sci*. **2023**, 622, 156946, https://doi.org/10.1016/j.apsusc.2023.156946.
4. Alamri S.; Rajhi A. A.; Tran N.; Senthil T. S.; Kumar Narukullapati B.; Mohanavel V. Tunable microwave absorption and shielding effectiveness in the nanocomposite of 3D hierarchical flower-like Co_3O_4 and rod-like polyindole. *J. Magn. Magn. Mater*. **2022**, 555, 169363, https://doi.org/10.1016/j.jmmm.2022.169363.
5. Ge J.; Cui Y.; Cai Y.; Qian J.; Liu L.; Meng F.; Wang F. 2D organic-α-$Co(OH)_2$ material and

derived Co/C composites with bifunctions of anti-corrosion and microwave absorption. *Compos. Part B: Engineering*. **2021**, 224, 109172, https://doi.org/10.1016/j.compositesb.2021.109172.

6. Zeng Z.; Xu D.; Li M.; Liu Z.; Xu R.; Liu D. Confined transformation of trifunctional $Co_2(OH)_2CO_3$ nanosheet assemblies into hollow porous Co@N-doped carbon spheres for efficient microwave absorption. *J. Colloid Interface Sci*. **2022**, 622, 625-636, https://doi.org/10.1016/j.jcis.2022.04.142.
7. Zhang D.; Yan C.; Zheng Y.; Han C.; Deng Y.; Yu J.; Zeng G.; Zhang H. Reduced graphene oxide wrapped 3D-ultrathin CoS_2 nanoflakes as an absorbing material with enhanced microwave absorption. *Prog Nat Sci-Mater*. **2022**, 32, 20-26, https://doi.org/10.1016/j.pnsc.2022.01.006
